# *Mucuna pruriens* (*Velvet bean*) Rescues Motor, Olfactory, Mitochondrial and Synaptic Impairment in *PINK1^B9^ Drosophila melanogaster* Genetic Model of Parkinson’s Disease

**DOI:** 10.1371/journal.pone.0110802

**Published:** 2014-10-23

**Authors:** Simone Poddighe, Francescaelena De Rose, Roberto Marotta, Roberta Ruffilli, Maura Fanti, Pietro Paolo Secci, Maria Cristina Mostallino, Maria Dolores Setzu, Maria Antonietta Zuncheddu, Ignazio Collu, Paolo Solla, Francesco Marrosu, Sanjay Kasture, Elio Acquas, Anna Liscia

**Affiliations:** 1 Department of Biomedical Sciences, University of Cagliari, Cagliari, Italy; 2 Department of Life and Environmental Sciences, University of Cagliari, Cagliari, Italy; 3 Electron Microscopy Lab, Nanochemistry Department, Istituto Italiano di Tecnologia, Genoa, Italy; 4 Institute of Neuroscience, National Research Council, Cagliari, Italy; 5 Department of Public Health, Clinical and Molecular Medicine, University of Cagliari, Cagliari, Italy; 6 Sanjivani College of Pharmaceutical Education and Research, Kopargaon, Ahmednagar, Maharashtra, India; 7 Center of Excellence for the Study of Neurobiology of Addiction, University of Cagliari, Cagliari, Italy; 8 National Institute of Neuroscience - INN, University of Cagliari, Cagliari, Italy; CSIR-Central Drug Research Institute, India

## Abstract

The fruit fly *Drosophila melanogaster* (*Dm*) mutant for PTEN-induced putative kinase 1 (*PINK1^B9^*) gene is a powerful tool to investigate physiopathology of Parkinson's disease (PD). Using *PINK1^B9^* mutant *Dm* we sought to explore the effects of *Mucuna pruriens* methanolic extract (*Mpe*), a L-Dopa-containing herbal remedy of PD. The effects of *Mpe* on *PINK1^B9^* mutants, supplied with standard diet to larvae and adults, were assayed on 3–6 (I), 10–15 (II) and 20–25 (III) days old flies. *Mpe* 0.1% significantly extended lifespan of *PINK1^B9^* and fully rescued olfactory response to 1-hexanol and improved climbing behavior of *PINK1^B9^* of all ages; in contrast, L-Dopa (0.01%, percentage at which it is present in *Mpe* 0.1%) ameliorated climbing of only *PINK1^B9^* flies of age step II. Transmission electron microscopy analysis of antennal lobes and thoracic ganglia of *PINK1^B9^* revealed that *Mpe* restored to wild type (WT) levels both T-bars and damaged mitochondria. Western blot analysis of whole brain showed that *Mpe*, but not L-Dopa on its own, restored bruchpilot (BRP) and tyrosine hydroxylase (TH) expression to age-matched WT control levels. These results highlight multiple sites of action of *Mpe*, suggesting that its effects cannot only depend upon its L-Dopa content and support the clinical observation of *Mpe* as an effective medication with intrinsic ability of delaying the onset of chronic L-Dopa-induced long-term motor complications. Overall, this study strengthens the relevance of using *PINK1^B9^ Dm* as a translational model to study the properties of *Mucuna pruriens* for PD treatment.

## Introduction

Parkinson’s disease (PD) is, after the Alzheimer’s disease, the second most prevalent neurodegenerative disease first affecting medulla oblongata, olfactory bulb and substantia nigra [1]. Loss of olfaction is a very consistent marker of PD occurring in 95% of patients early before the onset of motor symptoms [2]. Olfactory dysfunction is observed in PTEN-induced putative kinase 1 (*PINK1^B9^*) Parkinsonism, both in humans [3] and in animal models of PD [4]. The *Drosophila melanogaster (Dm) PINK1^B9^* mutant model recapitulates several of the essential features of PD [5] and has been used to study neuronal dysfunction and molecular aspects of neurodegeneration [6]. In particular, *PINK1^B9^* model provides major information regarding pathogenic molecular basis of early onset PD and mitochondrial dysfunction [5]. Accordingly, it was recently reported that *PINK1* mutation enhances mitochondrial stress-induced neurodegeneration in mice [7].

L-Dopa is the most effective symptomatic medication of PD and is still considered the gold standard in its treatment, although other drugs such as dopamine (DA) agonists, DA uptake and mono amino oxidase-B inhibitors are commonly used in the clinical management of PD patients [8] [9] [10]. Besides, other drugs such as adenosine A2A antagonists used as adjunct might be effective in the symptomatic treatment of PD [11]. In addition, the involvement of non-dopaminergic neurotransmitters such as noradrenaline, serotonin, glutamate, and acetylcholine in different brain areas like cortex, brainstem and basal ganglia has prompted many researchers to investigate the effects of non-dopaminergic drugs [12] indicating the involvement of multiple targets in treatment of PD.

Several reports on antiparkinsonian activity of *Mucuna pruriens* (*Mp*) [13] [14] endorse the use of *Mp* seeds in PD. In addition to L-Dopa, *Mp* seeds contain genistein and polyunsaturated fatty acids which support its antiparkinsonian and neuroprotective actions [15]. Furthermore, phytic acid, another *Mp* constituent with antioxidant and iron sequestrant activity, has been reported to suppress methyl-phenyl-tetrahydropyridine (MPTP) induced hydroxyl radical generation [16]. Hence, in view of multiple phytoconstituents supporting antiparkinsonian activity of *Mp*, the present study was aimed at verifying if *Mpe*’s ability to ameliorate symptoms in this PD model might be attributable to L-Dopa only or to the *Mp* extract as a whole in which L-Dopa is present along with other ingredients. On these bases we evaluated the antiparkinsonian profile of the standardized methanolic extract of the seeds of *Mp* (*Mpe*) on lifespan, climbing activity and olfactory function in *PINK1^B9^* as compared to either wild type (WT) and untreated *PINK1^B9^ Dm.* In addition, in order to gain mechanistic insights on the neuroprotective and neuro-rescue properties of *Mpe*, we also evaluated the expression of bruchpilot protein and tyrosine hydroxylase, as well as the morphology of presynaptic active zones and mitochondria in flies’antennal lobes, i.e. the olfactory bulbs-equivalent structure, and thoracic ganglia, of both WT as well as untreated and *Mpe*-treated *PINK1^B9^* mutants.

## Materials and Methods

### Fly Strains

For these experiments we used adult wild type (WT) Oregon-R (Oregon-R-C) and PTEN-induced putative kinase 1 *PINK1^B9^* (w[*] Pink1[B9]) mutant *Drosophila melanogaster* (*Dm*) males (from Bloomington Stock Center; Fly Base: http://flybase.bio.indiana.edu). After emergence from pupae, male WT and *PINK1^B9^* mutant flies were separated. WT and mutant flies were reared on a standard cornmeal-yeast-agar medium in controlled environmental conditions (24–25°C; 60% relative humidity; light/dark = 12/12 hours). In detail, four groups of mutant flies were reared on a standard medium supplemented with *Mucuna pruriens* methanolic extract (*Mpe*) (Batch no. FMPEX/2012060001; Natural Remedies Ltd., Bangalore, India). *PINK1^B9^* mutants were supplied with *Mpe* at different concentrations (0 (i.e. untreated *PINK1^B9^* mutants), 0.1, 1 and 10% w/w in their standard diet) both as larvae and adults (L^+^/A^+^). In addition, another group was reared on a standard medium supplemented with 0.01% (0.5 mM) L-Dopa (Sigma Aldrich, Milan, Italy), a percentage similar to that at which L-Dopa was supplemented with 0.1% *Mpe* [15]. The effects of *Mpe* were assayed at different age steps (I: 3–6; II: 10–15; III: 20–25 days old). A series of experiments on life span, using various concentrations of *Mpe* (see below in Survival curves) provided the basis for selecting the optimal concentration at which conduct the behavioral, morphological, and protein expression assessments. In particular, based on lifespan results, the olfaction behavior assessments, transmission electron microscopy (TEM) and western blot analyses were restricted to group II flies after 0.1% *Mpe* administration as L^+^/A^+^. Standard genetic procedures were used during the study.

### Survival curves

With the aim of selecting the optimal *Mpe’s* concentration, *Dm* were grown on standard diet supplemented with different concentrations of *Mpe* at 25°C. Cohorts of 40 flies (4 flies/tube) from each experimental group (i.e. WT, untreated and *Mpe-*treated *PINK1^B9^*) were monitored every 2 days for their survival. Mortality was analyzed using Kaplan-Meier survival curves and the statistical comparisons were made with a Gehan-Breslow-Wilcoxon test. Experiments were done in duplicate with the exception of those on WT, untreated mutants, 0.1% *Mpe*- and 0.01% L-Dopa-treated *PINK1^B9^* that were done in triplicate.

Each experiment was conducted with the appropriate control group (i.e. WT, untreated *PINK1^B9^* and treated *PINK1^B9^*).

### Climbing assay

The climbing assay (negative geotaxis assay) was used to assess locomotor ability [17]. Climbing data were obtained from groups I–III of untreated WT, untreated *PINK1^B9^* and, as L^+^/A^+^, 0.1, 1 and 10% *Mpe*- and 0.01% L-Dopa-treated *PINK1^B9^* mutants. Cohorts of 30 flies from each experimental group were subjected to the assay. Flies were placed individually in a vertically-positioned plastic tube (length 10 cm; diameter 1.5 cm) and tapped to the bottom. Climbing time was recorded upon crossing a line drawn at 6 cm from the bottom. The number of flies that could climb unto, or above, this line within 10 seconds was recorded and expressed as percentage of total flies. Data were expressed as average + SEM from at least three separate experiments. The statistical evaluation was made by two-way ANOVA (p<0.05) followed by HSD post-hoc test.

### Electroantennograms (EAGs) recordings

In vivo electroantennogram recordings (EAG) were performed following a previously described protocol [4]. Briefly, live adult WT *Dm* and untreated, *Mpe*- and L-Dopa-treated *PINK1^B9^* from group II (n = 12/each strain) were singly positioned under the view of an Olympus BX51WI light microscope (Olympus, Tokyo, Japan). Electrodes were silver wires inserted in glass capillaries filled with a saline solution (NaCl 150 mM).The recording glass electrode was positioned on the tip of the left antenna while the reference was pierced through the compound eye. The EAG signal was amplified with an AC/DC probe and then acquired with an IDAC-4 interface board (Syntech, Hilversum, NL). The antennae were constantly blown by a flow of charcoal purified and humidified air (speed 0.5 m/s) via a glass tube. Odor stimuli were administered by injecting a puff of purified air (0.5 s at 10 mL/s airflow) through the pipette using the stimulus delivery controller (Syntech, Hilversum, NL).

Odor stimuli were prepared in 3 step-dose concentration (0.01, 0.1, and 1% v/v) diluted in hexane. Odor stimulus, 1-hexanol, was chosen according to Fishilevich and Vosshall [20], for its well-known stimulant activity in *Dm*. Mean values of EAG amplitude were calculated and then analyzed by comparing the results obtained in untreated *PINK1^B9^*, *Mpe*- and L-Dopa-treated flies with matched WT. The significance of differences was tested by one-way ANOVA (followed by HSD post hoc test) with a threshold level of statistical significance set at p<0.05. EAG results are expressed as average values ± S.E.M and represented by histograms.

### Olfactory behavior

Free-walking bioassay was performed following the experimental procedures used by Dekker et al. [18]. In particular, group II WT, untreated *PINK1^B9^* and, as L^+^/A^+^, 0.1% *Mpe*- and 0.01% L-Dopa-treated *PINK1^B9^* mutants were given the opportunity to choose between vials containing water with or without odor. Two 4 mL glass vials were placed symmetrically in a large petri dish (arena) and then fitted with truncated pipette tips. The vials were filled with 300 µL of water with 0.25% Triton X (Sigma-Aldrich, Milan, Italy) with or without the odorant (0.1% (v/v) 1-hexanol; Sigma-Aldrich, Milan, Italy). As mentioned above, in order to allow detection of possible *Mpe’s* effects independently from the circuit, octopaminergic -for appetitive- and dopaminergic -for aversive stimuli [19], 1-hexanol was chosen, according to Fishilevich and Vosshall [20], because the mechanism(s) of olfactory transduction signal involve several glomeruli and complex neural pathways. Flies were starved for 8 hours prior to starting the experiments. These, done in triplicate, were performed in controlled environmental conditions (n = 12 bioassays/each experimental group of flies; n = 20 flies/arena). The assays lasted 18 hours, a streamlined range of time to overtake the possible influence of motor impairment in mutants. The dehydration of flies was prevented by placing a cotton ball with 3 mL of water in the arenas. Data obtained were expressed as average of percentages of flies reaching the 1-hexanol or water trap and statistically evaluated by one-way ANOVA (p<0.05) followed by HSD post-hoc test.

### Electron microscopy analysis

Group II WT, untreated *PINK1^B9^* and, as L^+^/A^+^, 0.1% *Mpe*-treated *PINK1^B9^* mutants were anesthetized using carbon dioxide and carefully decapitated. The brains and the thoracic ganglia, once rapidly removed, were fixed in a mixture of 2% glutaraldehyde and 2% paraformaldehyde in 0.1 M cacodylate buffer, washed several times in the same buffer, post-fixed in 1% osmium tetroxide in distilled water for 2 hours, and stained overnight at 4°C in an aqueous 0.5% uranyl acetate solution. After several washes in distilled water, the samples were dehydrated in a graded ethanol series, and embedded in SPURR resin. To identify the antennal lobes (ALs), semi-thin coronal sections of the whole brains were cut with a Leica EM UC6 ultramicrotome, stained with toluidine blue and observed with a Leica DM2700 P light microscope. Sections of about 70 nm corresponding to portions of the ALs and thoracic ganglia were cut with a diamond knife on a Leica EM UC6 ultramicrotome. Transmission electron microscopy (TEM) images were collected with a FEI Tecnai G2 F20 (FEI Company, The Netherlands) and a Jeol JEM 1011 (Jeol, Japan) electron microscopes, working respectively at an acceleration voltage of 80 and 100 kV, and recorded with a 1 and 2 Mp charge-coupled device (CCD) camera (Gatan BM Ultrascan and GatanOrius SC100, respectively). T-bars density (expressed as number of T-bars/m^2^) in both ALs and thoracic ganglia presynaptic boutons was assessed on a total of ten animals (three WT, three untreated *PINK1^B9^* and four 0.1% *Mpe*-treated *PINK1^B9^* mutants). 459 and 683 T-bars were randomly sampled respectively in the ALs and the thoracic ganglia on a total 496 non-overlapping micrographs at a final magnification of 6000, corresponding to a total sampled area of more than 6000 µm^2^. T-bars were unambiguously identified at presynaptic active zones by the presence of T-shaped electron-dense projections typically tethered by a large number of presynaptic vesicles.

The number of damaged mitochondria within ALs (expressed as percentage of the total number of mitochondria/sampled area) was evaluated in WT, untreated *PINK1^B9^* and 0.1% *Mpe*-treated *PINK1^B9^* mutants. More than 3000 mitochondria were randomly sampled on 191 non-overlapping micrographs at a final magnification of 4000, corresponding to a total sampled area of more than 5000 µm^2^. Damaged mitochondria were recognized for the presence of swollen external membrane, clearly fragmented cristae and inhomogeneous electron transparent mitochondrial matrix. The mean differences were tested using a two tailed *t*-test and a p<0.01 level was considered statistically significant.

### Protein extraction and western blot analysis

Group II WT, untreated *PINK1^B9^* and, as L^+^/A^+^, 0.1% *Mpe*- and 0.01% L-Dopa-treated *PINK1^B9^* mutants flies were collected and immediately stored at −80°C. Head lysate preparations of adult males were performed by homogenization in RIPA buffer (9.1 mmol/L dibasic sodium phosphate, 1.7 mmol/L monobasic sodium phosphate, 150 mmol/L sodium chloride, 1% Nonidet P-40, 0.5% sodium deoxycholate, 0.1% sodium dodecylsulfate [pH adjusted to 7.4]) containing fresh protease inhibitor cocktails (Sigma-Aldrich, St. Louis, MO, USA). Two centrifugations were performed at 4°C at 10,000 g for 15 minutes, before protein quantification by DC Protein assay (Biorad, Hercules, CA, USA). 20 µg of proteins were resolved by sodium dodecyl sulfate–polyacrylamide gel electrophoresis analysis using the mini-PROTEIN 3-electrophoresis module assembly (Biorad, Hercules, CA, USA) and then transferred to immobilon-polyvinylidenedifluoride membranes (Amersham Biosciences). The membranes were incubated with primary antibodies overnight at 4°C. Immune complexes were detected with horseradish peroxidase–conjugated secondary antibodies and chemiluminescence reagents (ECL, Amersham Biosciences) and visualized by Image Quant LAS 4000. Densitometric analysis was performed by Image Studio Lite software for quantitative assessment.

Primary antibodies used in this study were against nc82 (1∶100 dilution, DSHB); Tyrosine Hydroxylase (1∶1000 dilution, MAB 318 Merk Millipore); actin (1∶100, sc1616 Santa Cruz Biotechnology); Horseradish-peroxidase–conjugated secondary antibodies were purchased from Life Technologies. Statistical significance of the results was evaluated by one-way ANOVA (p<0.05) followed by a HSD post-hoc test.

## Results

### Effects of *Mucuna pruriens* and L-Dopa on life span of *PINK1^B9^* mutants

**Figure 1:**
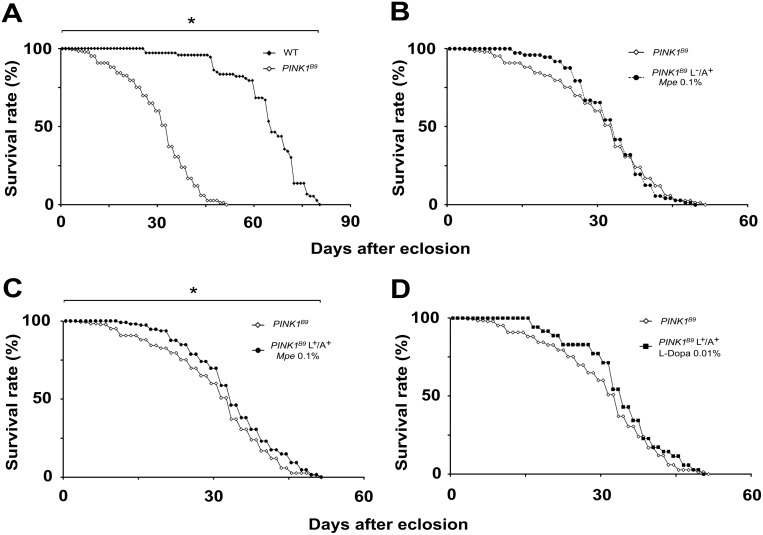
Effects of *Mpe* and L-Dopa on lifespan. (A): Lifespan, expressed as % survival rates, of wild type (WT) and *PINK1^B9^* flies. (B) and (C): Lifespan of *PINK1^B9^* treated with *Mucuna pruriens* extract (Mpe) 0.1%,only when adults (L^−^/A^+^) (panel B) or from their larval stage to the end of their life-cycle (L^+^/A^+^) (panel C), respectively, as compared to lifespan of untreated *PINK1^B9^* flies. (D): Lifespan of *PINK1^B9^* flies treated with L-Dopa (L^+^/A^+^) 0.01%. *indicates p<0.05 at Kaplan-Meier survival curves (Gehan-Breslow–Wilcoxon - GraphPad Prism 5.01) between WT and untreated *PINK1^B9^* (A) and between untreated *PINK1^B9^* and *PINK1^B9^* fed *Mpe* 0.1% (C).

As shown in Fig. 1A, in agreement with our previous report [4], *PINK1^B9^* mutants displayed a significantly shorter lifespan with respect to WT flies. To assess the ability of *Mpe* to affect lifespan of *PINK1^B9^* mutants, they were supplied *Mpe* at different concentrations (0 (untreated), 0.1, 1 and 10% w/w in their standard diet) both as adults only (L^−^/A^+^) (Fig. 1B and Fig. S1A), and as larvae and adults (L^+^/A^+^) (Fig. 1C and Fig. S1B). The effects of L-Dopa (supplied as L^+^/A^+^ (at the concentration, 0.01%, at which is present in the *Mpe* 0.1%) on life span of *PINK1^B9^* are also reported in Fig. 1D. The comparison between untreated and *Mpe*-treated *PINK1^B9^*, as shown by Kaplan-Meier survival curves, revealed a statistically significant effect of *Mpe* on lifespan of *PINK1^B9^* mutants only when L^+^/A^+^ flies were fed 0.1% *Mpe* (Fig. 1C, p<0.05 by Gehan-Breslow-Wilcoxon test). No effect was observed following the L-Dopa administration in L^+^/A^+^. As shown in Fig. S1A, no significant effects were detected in in L^−^/A^+^ flies, no matter the concentration tested, nor in L^+^/A^+^ flies fed 1% or 10% *Mpe* enriched standard diet (Fig. S1B).

### *Mucuna pruriens* rescues impaired climbing behavior of *PINK1^B9^* mutants

**Figure 2:**
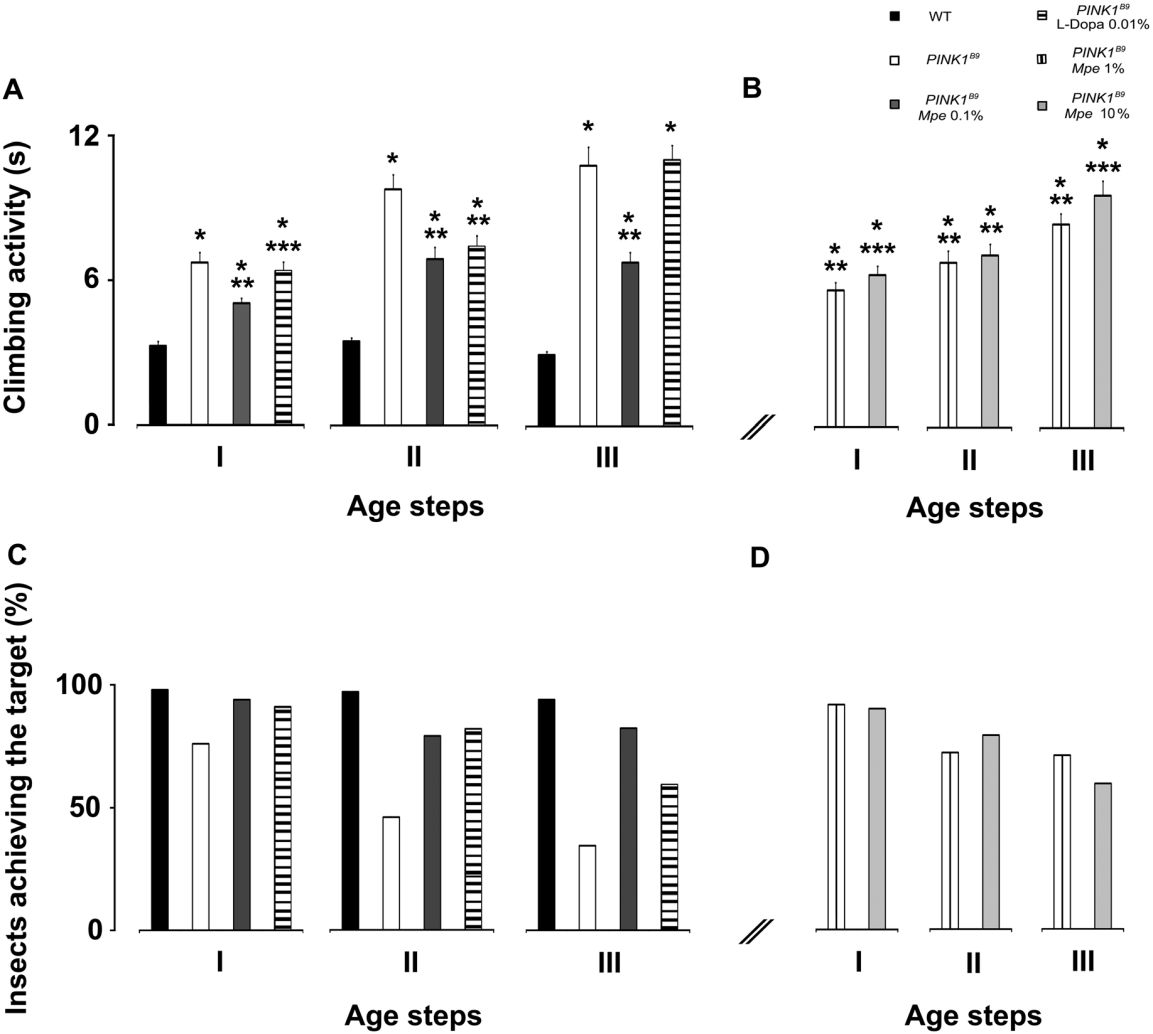
Effects of *Mpe* and L-Dopa on climbing activity. (A): Climbing activity of adult males wild-type (WT), untreated *PINK1^B9^*, *PINK1^B9^* treated with *Mucuna pruriens* extract (*Mpe*) 0.1% and *PINK1^B9^* treated with L-Dopa 0.01% (L-Dopa 0.01%). (B): Climbing activity of *PINK1^B9^* adult males treated with *Mpe* 1 and 10% as compared with WT and untreated *PINK1^B9^*. (A) and (B): Treatments were administered to flies from their larval stage to the end of their life-cycle (L^+^/A^+^) and their effects were assayed at three different age steps (I: 3–6; II: 10–15; III: 20–25 days) of flies’ life-span. Values are average + SEM. *indicates p<0.05 at two-way ANOVA followed by HSD post-hoc test as compared to WT; **indicates p<0.05 at two-way ANOVA followed by HSD post-hoc test as compared to *PINK1^B9^*; ***indicates p<0.05 at two-way ANOVA followed by HSD post-hoc test as compared to *PINK1^B9^ Mpe* 0.1%. (C) and (D): Percentages of adult males WT, *PINK1^B9^*, *Mpe* 0.1%, L-Dopa 0.01% (C) and *Mpe* 1 and 10% (D) that could climb unto, or above, the line drawn at 6 cm from the bottom of the tube within 10 seconds.

To investigate the locomotor ability the negative geotaxis assay, as described previously [17], was used. An impairment of climbing behavior was observed in untreated *PINK1^B9^* at different age steps (I: 3–6; II: 10–15; III: 20–25 days old) with a worsening trend with aging, while WT flies fulfilled the evaluation criterion without differences among age groups. As shown in Fig. 2A, the mutants took longer times to accomplish the task than the WT (p<0.001).

The *Mpe* 0.1% treatment significantly ameliorated the climbing activity in mutants and also reduced the worsening trend with aging although the score obtained by treated mutants still remained higher than that measured in WT. Interestingly, the climbing time of L-Dopa-treated mutants from groups I and III did not significantly differ with respect to age-matched untreated *PINK1^B9^*, the performance of only group II flies being significantly ameliorated.

As shown in Fig. 2B, L^+^/A^+^ 1% *Mpe*-treated mutants reached similar rescue of climbing activity as observed in 0.1% *Mpe*-treated ones only when tested at early ages (groups I and II). On the other hand, 10% *Mpe* administration failed to significantly ameliorate motor behavior in groups I and III with respect to untreated *PINK1^B9^* mutants, while a significant effect was detected in treated flies from group II. We also considered the percentages of flies that were able to complete the test and the results are depicted in histograms shown in Fig. 2C and D. In this respect, most of WTs of all age steps (97–98%) were able to complete the test, while only 76% of *PINK1^B9^* from group I, 46% from group II and 36% from group III accomplished it, showing a clear age-dependent worsening. Administration of 0.1% *Mpe*, as L^+^/A^+^, greatly rescued *PINK1^B9^* mutants (groups I–III) from motor impairment and restored to WT values the percentages (86–94%) of flies able to accomplish the task according to the evaluation criterion (10 sec). Furthermore, at variance with the above results, the effects of L-Dopa worsened over time. In particular, 0.01% L-Dopa administration determined a decrease of the number of flies able to complete the task showing a negative trend with aging. In fact, percentages of flies were 91%, 82% and 62%, in groups I, II and III, respectively.

### *Mucuna pruriens* and L-Dopa effects on the EAG amplitude

As expected, the olfactory stimulations of flies’ antennae elicited responses with the typical EAG wave form, i.e. a rapid depolarization followed by a slower recovery phase, ending with the hyperpolarized wave before complete reversal to the baseline.

**Figure 3:**
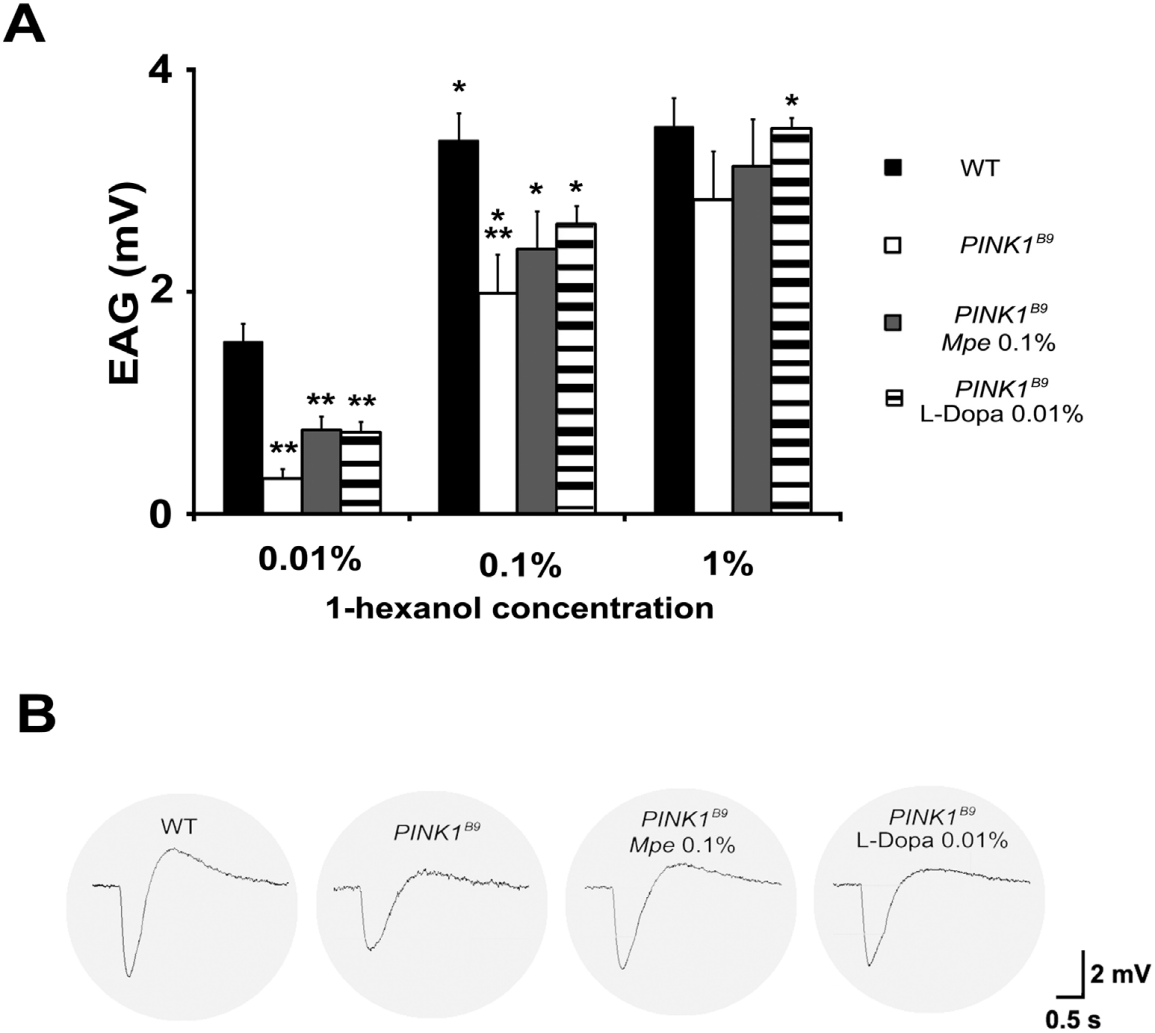
Electroantennogram responses to 1-hexanol. Histograms in (A) show the dose-response relationship and their differences in signal for olfactory stimulations in WT, untreated *PINK1^B9^* and in *Mpe* (0.1%)- and L-Dopa (0.01%)-treated *PINK1^B9^*, recorded in flies from group II. As odor stimuli, the 1-hexanol was administered in a 3-step dose from 0.01 to 1% in hexane. Values are average + SEM. *indicates p<0.05 at one-way ANOVA followed by HSD post hoc test as compared to the previous concentration of the stimulus. **indicates p<0.05 at one-way ANOVA followed by HSD post-hoc test as compared to WT. (B) Samples of EAGs recordings in response to 1-hexanol 0.1%.

The results, summarized in Fig. 3A and 3B, show the olfactory response to 1-hexanol (0.01, 0.1 and 1%) elicited in WT, untreated *PINK1^B9^*, 0.1% *Mpe*- and 0.01% L-Dopa-treated mutants from age group II. In details, the average EAG signal amplitudes evoked by stimuli were significantly lower in *PINK1^B9^* specimens in respect to WT thus substantially confirming data previously reported [4].

The stimulation with 1-hexanol at 1% did not elicit a significant increase in the EAG amplitude as compared with the stimulation at 0.1% in all strains of flies with the exception of mutants flies treated with L-Dopa 0.01%. This result indicates that at the highest odor concentrations (0.1 and 1%) a saturation of response was reached by all groups but by the L-Dopa treated mutants. Besides, we observed that the responses to stimuli in WTs elicited a greater hyperpolarized phase in the EAGs (Fig. 3B and S2).

Even if a positive trend in treated mutants exists in the signal amplitude in response to 1-hexanol, a statistical difference between untreated, *Mpe*- and L-Dopa-treated *PINK1^B9^* was not detected. The lowest odor concentration tested elicited a significantly higher response in WT as compared to the all strains of mutants (p<0.05). This difference shrank when the 0.1% concentration of odor was administered. A reduced response, although not statistically significant (p>0.05), was still detected in untreated mutants with respect to WTs (p<0.05), while treated flies showed on average an increased response with respect to untreated flies. The response measured in treated flies was therefore halfway between the highest of WTs and the lowest of untreated *PINK1^B9^*. Samples of EAGs responses are shown in Fig. 3B and Fig. S2.

### *Mucuna pruriens* rescues impaired olfactory behavior

**Figure 4:**
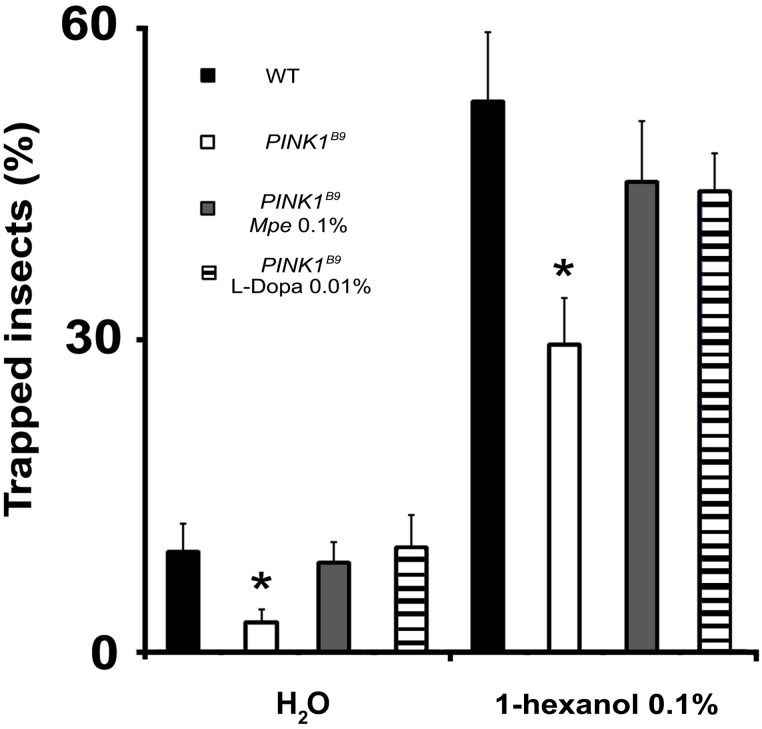
Effects of *Mpe* and L-Dopa on olfactory behavior. Responses to 1-hexanol 0.1% and water (H_2_O) of WT, untreated *PINK1^B9^* and in *Mpe* (0.1%)- and L-Dopa (0.01%)-treated *PINK1^B9^* flies. Values are average + SEM. *indicates p<0.05 at two-way ANOVA followed by HSD post hoc test as compared to WT, *PINK1^B9^ Mpe* 0.1%, *PINK1^B9^* L-Dopa 0.01%.

The olfactory behavior assay was restricted to flies of group II, by testing the responses to 1-hexanol (0.1% v/v) of WT, untreated *PINK1^B9^*, 0.1% *Mpe*- and 0.01% L-Dopa-treated, as L^+^/A^+^, *PINK1^B9^* mutants. As expected, the analysis of the result, shown in Fig. 4, confirmed the olfactory behavioral impairment in *PINK1^B9^* flies [4]. In fact, only 29.6±4.4% of mutant flies were odor-trapped, while the percentage of baited WT (52.9±6.6%) was significantly higher (*p*<0.004). *PINK1^B9^* flies treated with 0.1% *Mpe* and 0.01% L-Dopa were able to reach the stimuli as WT controls (p>0.05). In fact, percentages of trapped flies were 45.2±5.8% and 44.2±3.6% for 0.1% *Mpe*- and 0.01% L-Dopa-treated mutants, respectively. Similar results were obtained concerning the numbers of trapped flies in the blank bait (H_2_O) (*p*<0.05 between untreated *PINK1^B9^*with respect to WT, 0.1% *Mpe*- and 0.01% L-Dopa-treated mutants).

### Mucuna pruriens rescues loss of T-bars at active zones of presynaptic terminals and damaged mitochondria in the antennal lobes and thoracic ganglia

**Figure 5:**
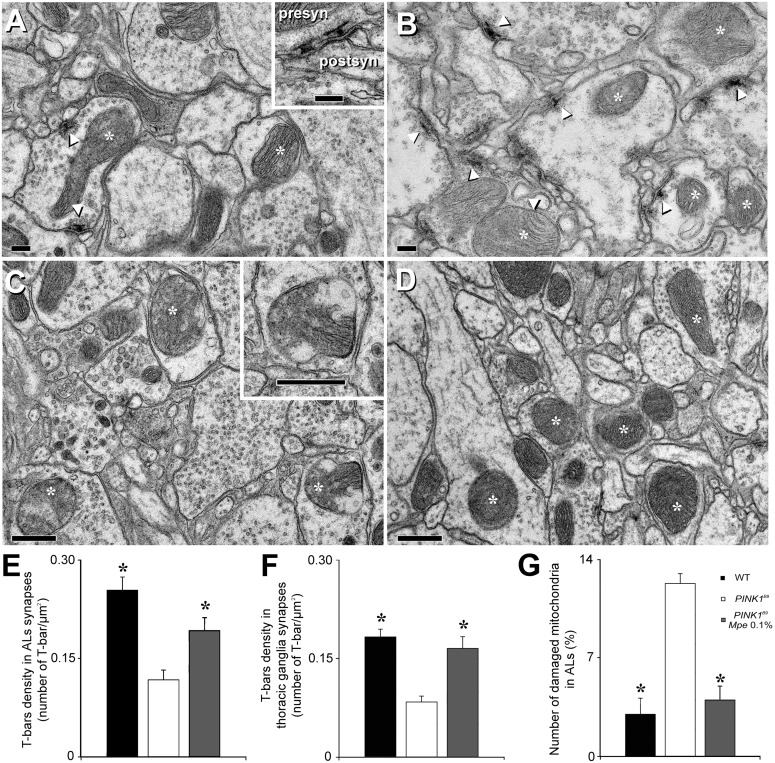
Effects of *Mpe* on T-bars and mitochondria in antennal lobes and thoracic ganglia. Transmission electron microscopy (TEM) images of T-bars and mitochondria inside antennal lobes (ALs) of wild type (WT), untreated *PINK1^B9^* and in *Mpe* (0.1%)-treated *PINK1^B9^* flies. (A): T-bars in a presynaptic bouton of *PINK1^B9^* ALs (arrowheads). Asterisks indicate mitochondria inside presynaptic boutons and neurites. Inset: high magnification of two T-bar in coronal section. (B): T-bars in presynaptic boutons of ALs of *PINK1^B9^ Mpe* 0.1% (arrowheads). Asterisks indicate mitochondria inside presynaptic boutons and neurites. (C): swelling on the external mitochondrial membrane (at high magnification in the inset) and mitochondrial cristae widely degenerated (asterisks) in ALs of *PINK1^B9^*. (D): Mitochondria of *PINK1^B9^ Mpe* 0.1% (asterisks). (E): Presynaptic T-bar density in ALs of WT, *PINK1^B9^* and *PINK1^B9^* 0.1% *Mpe* flies. Values are average + SEM. *indicates p<0.01 at two tailed *t*-test with respect to *PINK1^B9^*. (F): T-bar density in thoracic ganglia of WT, *PINK1^B9^* and *PINK1^B9^* 0.1% *Mpe* flies. Values are average + SEM. *indicates p<0.01 at two tailed *t*-test with respect to *PINK1^B9^*. (G): Percentages of damaged mitochondria in ALs of WT, *PINK1^B9^* and *PINK1^B9^* 0.1% *Mpe* flies. Values are average + SEM. *indicates p<0.01 at two tailed *t*-test with respect to *PINK1^B9^*. Abbreviations: postsyn: postsynaptic; presyn: presynaptic. Scale bars are 200 µm in A and B and 500 µm in C and D.

Transmission electron microscopy (TEM) analysis was restricted to flies of group II of untreated WT, untreated *PINK1^B9^* and 0.1% *Mpe*-treated, as L^+^/A^+^, *PINK1^B9^* mutants and results are shown in Fig. 5. A significant decrease of T-bars density was observed in the presynaptic bouton active zones of both ALs and thoracic ganglia of *PINK1^B9^* mutants with respect to WT controls (panels A, B, E and F). More importantly, a significant increase of T-bars density was detected in the ALs and thoracic ganglia of *PINK1^B9^* treated with 0.1% *Mpe*, as L^+^/A^+^, with respect to untreated *PINK1^B9^* (panels A, B, E and F). Moreover the number of damaged, swollen and with clearly fragmented cristae, mitochondria was significantly lower in presynaptic boutons of ALs of *PINK1^B9^* mutants treated with 0.1% *Mpe*, compared with untreated mutants (panels C, D and G).

### *Mucuna pruriens* and L-Dopa differentially affect whole brain bruchpilot (BRP) and tyrosine hydroxylase (TH) expression

**Figure 6:**
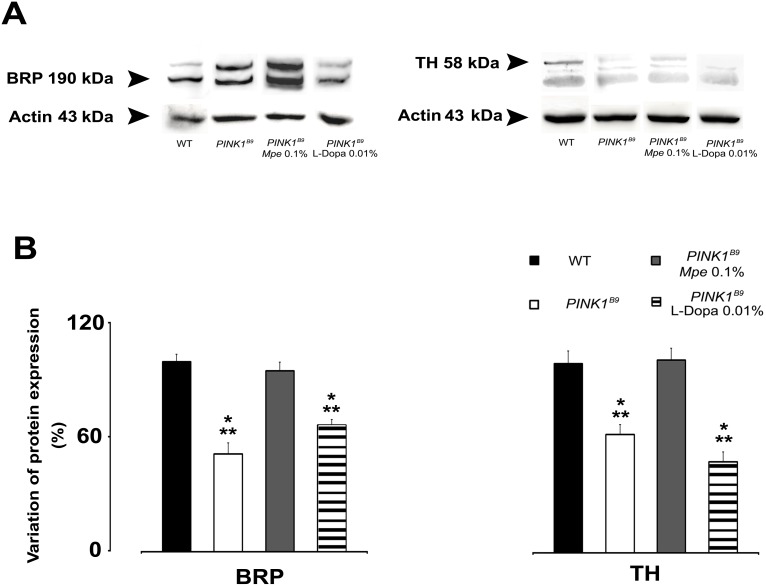
Effects of *Mpe* and L-Dopa on BRP and TH. (A): Representative western blot analysis of head homogenates from adult wild type (WT), untreated *PINK1^B9^* and in *Mpe* (0.1%)- and L-Dopa (0.01%)-treated *PINK1^B9^* flies showing labeled bands of Bruchpilot protein (BRP), of Tyrosine hydroxylase (TH) and of the loading control actin (from top to bottom). (B): Percentage of protein expression variation of BRP and TH in WT, untreated *PINK1^B9^* and in *Mpe* (0.1%)- and L-Dopa (0.01%)-treated *PINK1^B9^* flies. Values are average + SEM. *indicates p<0.05 at one-way ANOVA with respect to WT; **indicates p<0.05 at one-way ANOVA (HSD post-hoc test) with respect to *PINK1^B9^ Mpe* 0.1%.

Fig. 6 shows the results of western blot analysis of whole brain expression of BRP and TH of flies of group II WT, untreated *PINK1^B9^* 0.1% *Mpe*- and 0.01% L-Dopa-treated, as L^+^/A^+^, *PINK1^B9^* mutants. As shown in Fig. 6A, the expression of BRP and TH in untreated *PINK1^B9^* mutants was significantly lower (p<0.05) than in WT. Diet supply of 0.1% *Mpe* to *PINK1^B9^* mutants significantly recovered BRP and TH expression to WT controls levels (p<0.05) and these values did not differ statistically from those of WT. Notably, BRP and TH expression in *PINK1^B9^* mutants fed 0.01% L-Dopa resulted similar to BRP and TH expression in untreated *PINK1^B9^* mutants. ANOVA also revealed that both BRP and TH expression resulted statistically different as compared to their expression of both WT and *PINK1^B9^* mutants fed 0.1% *Mpe*.

## Discussion

This study was aimed at characterizing the effects of the standardized extract of *Mucuna pruriens* seeds, known for possible neuroprotective effects in neurotoxin-induced models of PD [21] [22] and reduced risk of dyskinesias [14], in a genetic fly model of PD, the *PINK1^B9^* mutant *Dm* [23]. Notably, mutations at *PINK1* gene model a number of features of early onset PD such as cell energy maintenance [24] and compromised olfactory and mitochondrial function [4] enabling in-depth investigations into physiopathology of *PINK1^B9^*-related molecular, morphological and functional bases of PD. The present results show that addition of 0.1% *Mpe* to the feeding medium of *PINK1^B9^* mutants significantly a) improved climbing ability and olfaction, b) rescued compromised T-bars density and damaged mitochondria in the ALs and thoracic ganglia, c) restored to WT control values the expression of BRP and TH proteins. Moreover, these results suggest that *Mpe* is an effective medication with intrinsic ability of delaying the onset of chronic L-Dopa-induced long-term motor complications (Fig. 2A and B).

These findings are in general agreement with previous studies reporting antiparkinsonian activity of *Mp* [14] [25] associated with reduced risk of dyskinesias, both in the clinical [26] and in the experimental [14] setting, and suggest that its antiparkinsonian effects may be due to components other than L-Dopa or that its components might have L-Dopa-enhancing effects [25] [26] [27] on one hand, as well as L-Dopa-induced dyskinesias (LID)-preventive effects, on the other.

Intriguingly, *PINK1^B9^* mutations have been linked to both autosomal recessive and sporadic forms of PD and, given the role of *PINK1*-*parkin* pathway in regulating mitochondrial function, our findings highlight its role as a potential target for the described actions of *Mpe* [30] on mitochondria. This interpretation finds further support in the observation of mitochondrial stress-dependent neurodegeneration [7] and dysfunction in *PINK1* knock-out mice [31].

A large body of literature documents that mutations of *PINK1* gene are associated with mitochondrial dysfunction. In particular, complex I deficiency [24] [32] has been characterized as a mechanism of energy balance failure [33] resulting also in dramatic loss of dopaminergic neurons [34]. Although in the present study we did not attempt any direct measurement of mitochondrial energy impairment, this dysfunction was indirectly determined by assessing the number of damaged, swollen and with clearly fragmented cristae, mitochondria and we found that 0.1% *Mpe* administration could dramatically recover their morphology to that of WT controls (Fig. 5). This indicates that *Mpe* may play beneficial actions by interfering with the mechanisms responsible of energy production [24] or linked to maintenance of membrane gradients as well as to protection against the raise of reactive oxygen species within mitochondria [16]. In this regard, it is intriguing to observe that *Mp* has antioxidant properties [35] and it was suggested that its “rescue” properties may be due to increased complex-I activity and presence of nicotinamide adenine dinucleotide and coenzyme Q-10 [25]. This interpretation is also supported by the observation that also enhancement of nucleotide production, by feeding *PINK1* mutant *Dm* with folic acid, results in rescued loss of mitochondrial mass and function [31]. Thus, on the basis of these reports and of our results it seems possible to speculate that *Mpe* administration interferes with the pathway regarding the mitochondrial rescue from oxidative stress but not on the complex apoptosis mechanism. In fact, the clock of the end of the life is not modified as also suggested by the results regarding the effect of *Mpe* on life span according to which the amelioration is slight, albeit significant. In agreement with Poddighe et al. [4], *PINK1^B9^* mutants showed steeper slope life span curves and overall shortened lifespan with respect to WT. *Mpe* significantly attenuated these conditions only when administered to L^+^/A^+^ at 0.1%, but not when administered to adults only (L^−^/A^+^) no matter the concentration tested (Fig. S1A), nor when administered with 0.01% L-Dopa. These results can be explained by taking into account that in *Drosophila* the cluster of neurons is manly conserved from larval to adult stage [28]. Conversely in L^+^/A^+^ mutants treated at the highest concentration administered (1–10%) even if not significant, a worsening trend was observed (Fig. S1B). The effects of *Mpe* on flies’ lifespan resemble those of the *Mpe* component, nicotine, described in a *Drosophila* autosomal recessive-juvenile model of parkinsonism [29].

In addition to the observed rescue of damaged mitochondria in *PINK1^B9^* mutants treated with 0.1% *Mpe*, we observed that this treatment significantly recovered the expression of BRP and the reduction of T-bars density in both *PINK1^B9^* ALs and thoracic ganglia, strengthening the tenet that BRP is crucial for the correct formation of T-bars at active zones [36]. Mutation-induced mitochondrial degeneration may also have led to the observed diminished expression of BRP, known to be critical also for neurotransmitter release [37] [38]. Accordingly, *PINK1^B9^* mutants show degeneration of flight muscle and of dopaminergic neurons accompanied by locomotive defects [23] [39] [40]. Humphrey et al. [40] also showed that climbing deficit is related to dysfunction of dopaminergic cells and we found that *PINK1^B9^* mutants also showed compromised motor capabilities as assessed by climbing behavior (Fig. 2). Hence, *Mpe-*increased expression of BRP may have increased the ability to release neurotransmitters that would result in improved locomotion, as suggested by Yellman et al. [41]. Therefore, our data suggest that the effects of *Mpe* treatment on BRP expression, climbing and T-bars in *PINK1^B9^* mutants may represent the convergence toward an unified mechanism grounded on mitochondria functional rescue.

Olfactory dysfunction is a clinical early non-motor symptom of PD [3] and, accordingly, we observed loss of olfaction in *PINK1^B9^* mutant *Dm* [4]. The physiopathology of olfactory dysfunction is not known. However, many studies have suggested involvement of dopaminergic system [42] [43]. In our investigation we observed improved olfactory responsiveness underlined by both behavioral and electrophysiological experiments.

It is interesting to observe that the shape of EAG responses recorded in the WT revealed a dose-related hyperpolarizing part (Fig. S2). This observation seems in accordance with the stimulating power of 1-hexanol that is reported to involve both the appetitive and the aversive stimuli [19]. The EAG represent the summed activity of all antennal sensory neurons involved in stimulation. This activity can result in the EAG recordings in a depolarization and/or hyperpolarization signal, that is elicited according to the stimulating effect of the odor tested as well as of its concentration. In details, in WT strain, the rapid depolarization is followed by a slow recovery phase at the lower concentration (0.01%) while at the highest concentration (1%), a greater hyperpolarizing phase was recorded. This phase could represent the activation of a pool of receptors that hyperpolarize when stimulated at this high concentration. With regards to this, a similar response was not present in untreated *PINK1^B9^* (Fig. S2). Future electrophysiological analysis of the olfactory response should take into account both shape and amplitude whose variations might be a promising tool to study peripheral olfactory responses. Furthermore, our behavioural results show that *PINK1* ^B9^ mutants have a decreased responsiveness to 1-hexanol and water (Fig. 4) that reveals an impairment of also other chemoreceptors such as hygroreceptors [44]. In other words the mutants seem to present a general sensory impairment.

In agreement with study by Katzenschlager and Lees [45], suggesting a possible association between olfaction, increased TH and dopamine in the olfactory bulbs, we observed that *PINK1^B9^* mutation-dependent impairment of olfaction behavior and whole brain TH expression were improved by *Mpe* treatment (Figs. 4 and 6, respectively). Surprisingly, L-Dopa administration on its own failed to recover TH expression to WT controls levels. However, since our analysis was done in whole brain homogenates, if analysis was restricted to the ALs, the homologous structures of human olfactory bulbs, we cannot exclude the possibility that L-Dopa would have brought different results.

The physiopathology of LID is still largely unknown and LID has consistently been related to excessive DA release [46]. Furthermore, in parkinsonian non-human primates [47], L-Dopa produces LID without enhancing striatal DA release. Interestingly, Katzenschlager et al. [26] observed a reduced severity of dyskinesias after *Mp* as compared to levodopa/carbidopa combination and an increased DDC expression associated with LID has been reported in rats [48]. This intriguing prospective remains to be fully demonstrated in the *Dm* mutant model. In conclusion, our study confirms in this translational model the validity of *Mucuna pruriens* as a valuable approach for PD treatment, discloses mechanistic insights at the basis of its effects and confirms the use of *PINK1^B9^ Dm* as a model of PD that fulfills the required face, construct and predictive validity criteria to follow up on these investigations.

## Supporting Information

Figure S1 **Effects of** ***Mpe*** **administered at different concentration on lifespan.** (A): Lifespan, expressed as % survival rates of untreated and *Mpe*-treated *PINK1^B9^* at the 4 dose-step tested: 0, 0.1, 1 and 10% (w/w) only when adults (L^−^/A^+^). (B): lifespan of untreated and *Mpe*-treated *PINK1^B9^* at the 4 dose-step tested: 0, 0.1, 1 and 10% (w/w) only when adults (L^+^/A^−^).
(TIF)

Figure S2 **EAGs samples.** Dose-response relationships for olfactory stimulations in WT and *PINK1^B9^* adult flies and their differences in signal amplitude and shape.
(TIF)
