# Expression of Concern: *Mucuna pruriens* (*Velvet bean*) Rescues Motor, Olfactory, Mitochondrial and Synaptic Impairment in *PINK1^B9^ Drosophila melanogaster* Genetic Model of Parkinson’s Disease

**DOI:** 10.1371/journal.pone.0231371

**Published:** 2020-04-01

**Authors:** The PLOS ONE Editors

Following the publication of this article [1], concerns were raised about the reporting of the western blot data presented in Fig 6A.

The bands presented in Fig 6A appear to have been spliced from the underlying gel and arranged into Fig 6A as presented in the published article. The preparation and presentation of the bands raises concerns regarding the reliability of the data presented in the published figure. The authors have informed the journal that the uncropped blots for Fig 6A are no longer available.

The authors have indicated that the original data underlying other results from this study are also not available. In light of this, the article is not in compliance with the journal’s Data Availability policy in place at the time of the article’s publication.

The *PLOS ONE* Editors issue this Expression of Concern to notify readers of the unresolved concerns pertaining to the reliability of the data presented in Fig 6A and the unavailability of underlying data to support the results presented in the article.
