# Supplementary figures and images for: Mucuna pruriens (Velvet bean) Rescues Motor, Olfactory, Mitochondrial and Synaptic Impairment in PINK1B9 Drosophila melanogaster Genetic Model of Parkinson’s Disease
Source: PLoS One. 2014 Oct 23;9(10):e110802. doi: 10.1371/journal.pone.0110802 (PMC4207759; doi:10.1371/journal.pone.0110802)

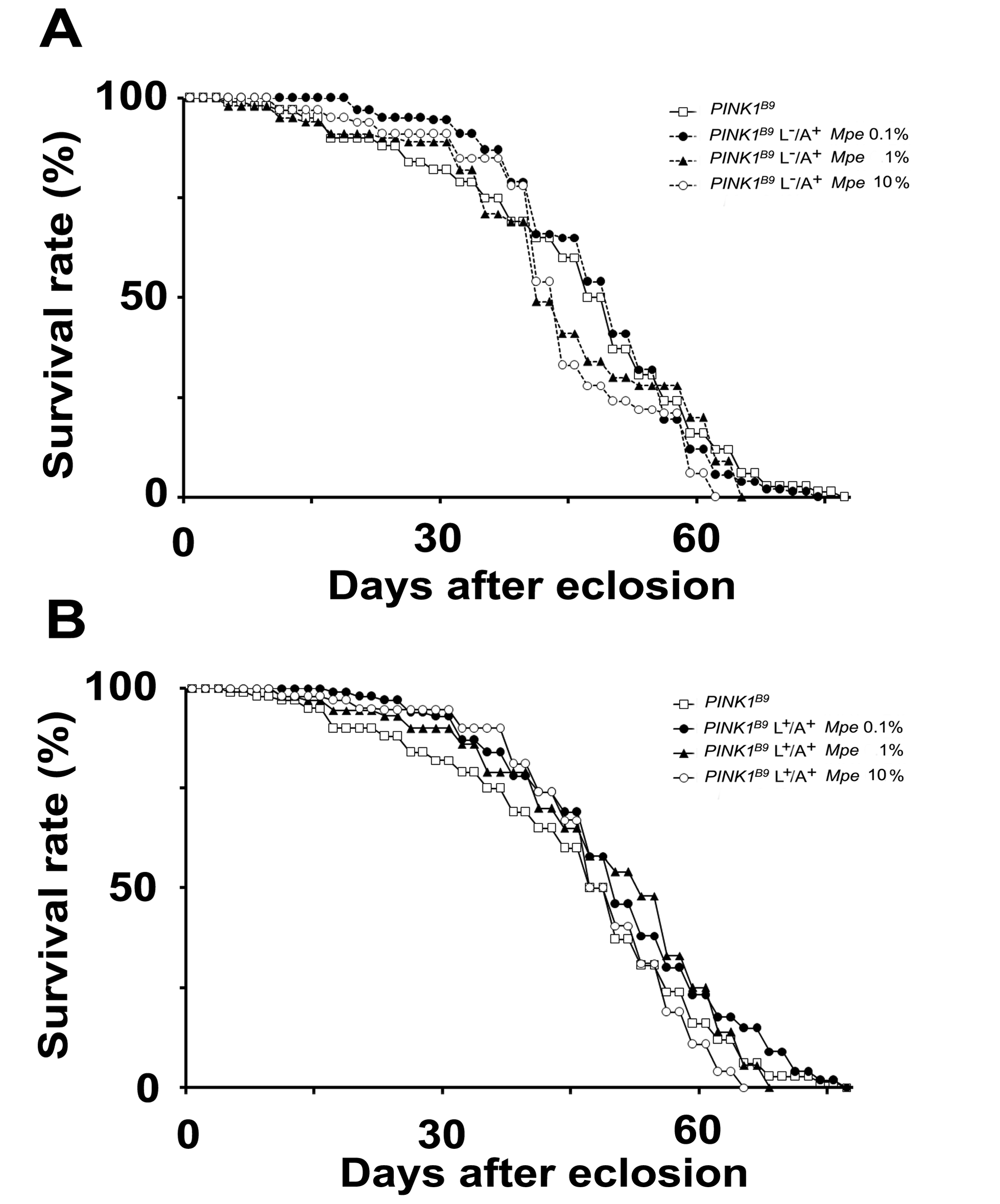

Supplement: Figure S1 — Effects of Mpe administered at different concentration on lifespan. (A): Lifespan, expressed as % survival rates of untreated and Mpe-treated PINK1B9 at the 4 dose-step tested: 0, 0.1, 1 and 10% (w/w) only when adults (L−/A+). (B): lifespan of untreated and Mpe-treated PINK1B9 at the 4 dose-step tested: 0, 0.1, 1 and 10% (w/w) only when adults (L+/A−). (TIF) [file pone.0110802.s001.tif]

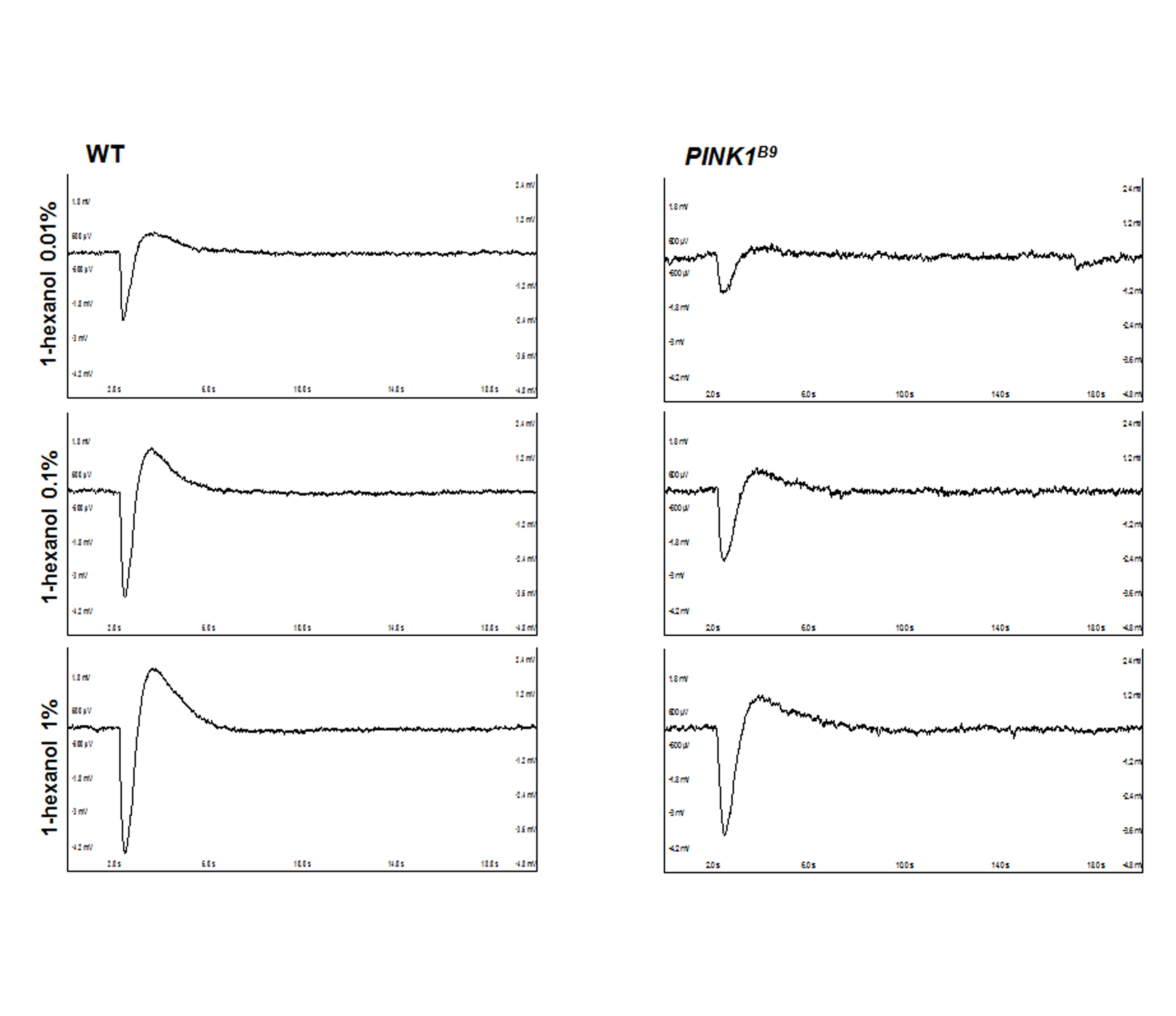

Supplement: Figure S2 — EAGs samples. Dose-response relationships for olfactory stimulations in WT and PINK1B9 adult flies and their differences in signal amplitude and shape. (TIF) [file pone.0110802.s002.tif]
